# Skin cancer in solid organ transplant recipients: still an open problem

**DOI:** 10.3389/fmed.2023.1189680

**Published:** 2023-04-21

**Authors:** Simona Granata, Gianpaolo Tessari, Giovanni Stallone, Gianluigi Zaza

**Affiliations:** ^1^Renal, Dialysis and Transplantation Unit, Department of Medical and Surgical Sciences, University of Foggia, Foggia, Italy; ^2^Section of Dermatology and Venereology, Department of Medicine, University of Verona, Verona, Italy

**Keywords:** solid organ transplantation, skin cancer, immunosuppression, cutaneous squamous cell carcinomas, basal cell carcinomas

## Abstract

In the last two decades, the optimization of organ preservation and surgical techniques, and the personalized immunosuppression have reduced the rate of acute rejections and early post-transplant complications. However, long-term graft survival rates have not improved over time, and evidence suggest a role of chronic calcineurin inhibitor toxicity in this failure. Solid organ transplant recipients may develop chronic dysfunction/damage and several comorbidities, including post-transplant malignancies. Skin cancers, mostly non-melanoma skin cancers (squamous cell carcinoma and basal cell carcinoma), are the most common malignancies in Caucasian solid organ transplant recipients. Several factors, together with immunosuppression, may contribute to the susceptibility for skin cancers which, although often treatable, could be associated with a much higher mortality rate than in the general population. The rapid identification and treatment (including reduction of immunosuppression and early surgical treatments) have an important role to avoid an aggressive behavior of these malignancies. Organ transplant recipients with a history of skin cancer should be followed closely for developing new and metastatic lesions. Additionally, patient education on the daily use of sun-protective measures and the recognition of the early signs (self-diagnosis) of coetaneous malignancies are useful preventive measures. Finally, clinicians should make themselves aware of the problem and build, in every clinical follow-up center, collaborative network involving transplant clinicians, dermatologists and surgeons who should work together to easily identify and rapidly treat these complications.

In this review, we discuss the current literature regarding the epidemiology, risk factors, diagnosis, preventive strategies and treatments of skin cancer in organ transplantation.

## 1. Introduction

In the last 20 years, improvements in organ preservation, optimization of surgical techniques, progress in post-operative care, and the introduction of more effective immunosuppressive drugs have led to significant advances in long-term graft and patient survival in organ transplantation. However, most transplant recipients experience systemic complications, often induced by over-immunosuppression, including malignancies (1–6).

Skin cancers, mostly non-melanoma skin cancers (NMSC) (squamous cell carcinoma and basal cell carcinoma), represent the most common form of cancer in Caucasian solid organ transplant recipients, with a continuing increase in incidence worldwide (7, 8). Several factors, in addition to immunosuppression, may contribute to the skin cancer risk (including genetic background, older age, male sex, fair skin type, and ultraviolet exposure) and, even though frequently treatable, these malignancies may significantly increase morbidity and mortality of this fragile and complex patients’ population (7, 8).

Therefore, an early diagnosis and treatment of these skin lesions may improve post-transplant outcomes. To this purpose, transplant clinicians and researchers worldwide are increasing research study protocols based on a multidisciplinary approach (including dermatologists, biologists, pharmacologists, surgeons).

Furthermore, clinicians in charge of these patients should be aware of the high risk of skin cancer development after solid organ transplantation and acquire basic knowledge of its epidemiology, risk factors, diagnosis, preventive strategies and treatments.

## 2. Epidemiology

### 2.1. Actinic keratosis

Actinic keratoses (AKs) are cutaneous neoplasm consisting of proliferation of cytological aberrant epidermal keratinocytes, that develop in response to prolonged exposure to ultraviolet radiation. AKs are now considered the initial lesion in a disease continuum that may progress to squamous cell carcinoma (SCC) (9, 10). AKs are common in solid organ transplant recipients, up to 80% of them may present AKs, and approximately 30% of patients may have 5 or more AKs. AKs are a strong risk factor for SCC, both in the general population and in transplant recipients (9, 10).

### 2.2. Non melanoma skin cancers

Non melanoma skin cancers (NMSC), and especially basal cell carcinoma (BCC) and squamous cell carcinoma (SCC), are the most common cancers observed in solid organ transplant recipients. The cumulative incidence of NMSC is related to geographic latitude, skin type, and immunosuppressive therapies. Australia has the highest incidence of NMSC in organ transplant recipients, with a 1-year, 10-year, and 20-year incidence of 7, 45, and 82%, respectively. In the United States, the 10-year incidence of NMSC is about 35%; lower rates (10–15%) are reported in Southern Europe (11–13). In Italy, it has been reported a 10-year incidence of NMSC of 10%, with a 3-year incidence of a second NMSC of 32% (14, 15). NMSCs occur after a median of 8 years after transplantation, except for patients transplanted after the age of 60, that may develop a NMSC after 3–5 years (16, 17). In transplanted patients, the standardized incidence ratio (SIR) of SCC is between 65 to 250, and SIR of BCC is 10, with an inversion of BCC/SCC ratio (16, 17). BCC is more common in the first years after transplantation, and the risk increases in a linear fashion in the post-transplant time, but the risk of SCC increases in an exponential manner (18).

### 2.3. Merkel cell carcinoma

Merkel cell carcinoma (MCC) is a rare, aggressive primary neuroendocrine skin cancer associated with infection with Merkel cell polyomavirus (MCV). In US transplant recipients, the overall incidence rate of MCC was 12.8 cases per 100,000 person-years, with a 24-fold elevation in risk, if compared with the general population. Seventy-three per cent of patients with MCC were older than 50 years at time of transplantation. MCC incidence was 70% higher in males, and 91% of MCC cases were in Caucasian patients (19, 20).

### 2.4. Melanoma

Melanoma is rare among renal transplant recipients. In a cohort of 105,174 renal transplant recipients, from the United States Renal Data System (USRDS), followed up in the years 2004–2012, only 448 patients (0.4%) developed a melanoma. The age-standardized relative rate of melanoma, if compared with the general population was 4.9 (21). In Italy, no excess risk of melanoma if compared with the general population has been described (22). Immunosuppressive drugs may enhance the development of nevi, due to reduced immunosurveillance and to local increased expression of α-melanocyte-stimulating hormone receptors (23). The mean total count of benign nevi has been found to be significantly higher in renal transplant recipients, if compared with sex and age-matched healthy controls, and it was related to the duration of immunosuppression (24). Piaserico et al., described the eruption of up to 500 nevi in a 16-year-old boy 6 months after kidney transplantation. Most of these nevi disappeared after chronic rejection and withdrawal of immunosuppression (25). Other rare cutaneous neoplasms among transplant recipients, including B and T primary cutaneous lymphomas, atypical fibroxanthoma, verrucous carcinomas, and leiomyosarcoma, have been reported (26–28).

## 3. Risk factors

### 3.1. Immunosuppressive drugs

The type of immunosuppressive drug, the duration and the dosage of immunosuppression correlate with skin cancer risk (29–31). Azathioprine can also directly induce UVA-mediated DNA mutagenesis (32). SCC is the most frequent NMSC among solid organ transplant recipients receiving cyclosporine (33, 34). Calcineurin inhibition inhibits nucleotide excision repair, which is the exclusive repair mechanism for the two most common UV-mediated types of DNA damage leading to photo carcinogenesis: cyclobutane pyrimidine dimers (CPD) and pyrimidine-6,4-pyrimidone photoproducts (6-4PP). Increased production of transforming growth factor-beta (TGF-b), potentiation of the oncogene ATF3, decreased apoptosis following UVB, and interruption of nuclear factor of activated T-cells (NFAT) have also been demonstrated with cyclosporine, each increasing potential for malignancy (35, 36).

Tacrolimus-based regimens seem to reduce the incidence of NMSC, but with no general agreement. A retrospective analysis of over 35,000 American solid organ transplant recipients revealed a 35% risk reduction for NMSC associated with tacrolimus, and cyclosporine having a risk ratio of 1 (37). Two large studies failed to detect a difference in NMSC risk between tacrolimus and cyclosporine (38, 39). However, contradicting evidence also exists, suggesting for tacrolimus a 2-to 4-fold increased risk of NMSC compared with cyclosporine (40–42). Mycophenolate Mofetil (MMF) seems to have less effect on photo-carcinogenesis than azathioprine and calcineurin inhibitors. A study demonstrated a 57% reduction in SCC risk in patients treated with MMF, if compared with patients receiving other immunosuppressive drugs (43). In liver transplant recipients, changing therapy from calcineurin inhibitors to MMF resulted in significantly lower rates of NMSC (hazard ratio 0.23) (44). Conversely, two retrospective analyses of heart transplant recipients failed to detect lower rates of malignancies in patients receiving MMF, if compared with mammalian target or rapamycin (mTOR) inhibitors and azathioprine, respectively (45).

A recent meta-analysis reviewed the impact of sirolimus on cancer risk (46). When analyzing the whole cohort, the cumulative incidence of NMSC was lower in the sirolimus group if compared with a control group of patients treated with traditional immunosuppressive drugs, with an adjusted hazard ratio of 0.60. However, patients receiving sirolimus had an increased proportion of hematological malignancies (0.64% vs. 0.19%), and a similar incidence of non-cutaneous cancers (*p* = 0.65). Sirolimus use was associated with a 43% increased risk of death (adjusted hazard ratio 1.43). Cancer-related mortality was about 0.20% in both groups. Patients receiving sirolimus presented a higher proportion of death from infection (0.58% vs. 0.15%) and cardiovascular disease (1.28% vs. 0.54%). In a sub-analysis, the protective effect of sirolimus against cancer was significant only among patients who converted from traditional therapy to sirolimus based therapy. These benefits should be balanced against the increased risk of cardiovascular and infection-related mortality (47–49).

### 3.2. Human papillomavirus (HPV) infection

Although the role of oncogenic human alpha-papillomaviruses in the development of mucosal carcinomas in different body sites (e.g., cervix, anus, oropharynx) is fully recognized, a possible role for human papillomavirus in actinic keratosis and squamous cell carcinoma (SCC) has been described (50–52).

Cutaneous SCCs are more frequent and more aggressive in solid organ transplant recipients compared with the general population (11). High prevalence (65–81%) and broad spectrum of HPV DNA types have been reported in premalignant skin lesions and in skin cancers of transplant recipients (53, 54).

A study by Reuschenbach et al., described a high-risk of HPV infection in 46.2% of the SCC of renal transplant recipients compared with 23.5% in the not transplanted control group (55). Moreover, a high prevalence of HPV was detected (by DNA and antibodies) in eyebrow hairs of kidney transplant recipients both with SCC and without SCC (56).

Although this interesting evidence, the exact contribution of HPV to the development of skin cancer in transplant recipients is still largely unrecognized.

### 3.3. UV exposure

Cumulative UV exposure appears to be a primary carcinogen; three-quarters of renal transplant NMSC cases occur on photo-exposed skin sites such as head, neck, upper limbs, and lower limbs (57). The mechanisms of photo carcinogenesis include direct DNA damage, UV effects on host immunity, and synergism with other drug-affected molecular pathways. Other strong risk factors for NMSC in solid organ transplant recipients include age at transplantation older than 50, fair skin, and male gender. In South African and in Asian studies, the incidence of NMSC was higher among Caucasian transplant recipients if compared with patients with African and Asian descent (58).

### 3.4. Other risk factors

Re-transplantation in patients with a previous SCC enhances the risk of future SCC (5, 45). Patients with life-saving organs such as heart, kidney-pancreas, and lung require intense immunosuppression and are about 2–3 times at higher risk of NMSC, if compared with kidney transplant recipients (30). Cumulative NMSC incidence following heart transplantation was 31% at 5 years and 43% at 10 years in an Australian cohort (30). Liver transplantation may have the lowest incidence of NMSC, possibly because the liver is less immunogenic and requires a minimal long-term immunosuppressive therapy (9, 59). Other risk factors for NMSC include personal history of NMSC, dialysis duration, smoking, and prophylaxis of fungal infections with voriconazole, possibly due to its photosensitive activity (11, 57, 58).

## 4. Treatment

Surgical excision of the entire lesion with adequate margins of clinically normal tissue around the tumor is the procedure of choice for any NMSC, especially in immunosuppressed patients, and exhaustive Italian guidelines have been recently published (60). NMSCs respond well to radiotherapy, and patients with unresectable carcinomas can expect excellent local control rates exceeding 90–95%. Adjuvant radiotherapy significantly reduces the risk of recurrence in SCC of the head and neck, and especially of the lower lip (hazard ratio 0.08). As reported in general population, complete lymph node dissection and postoperative radiotherapy could provide excellent freedom from locoregional relapse (61).

Moreover, as largely discussed in consensus expert panels, a recommended mild reduction in transplant-associated immunosuppression once multiple skin cancers developed per year or with individual high-risk skin cancers is largely suggested. Moderate reductions were considered appropriate when patients experienced >25 skin cancers per year or for skin cancers with a 3-year mortality risk of 10%. Severe reductions were considered for life-threatening skin cancers (62).

Immunosuppression can be modified by a decrease in the dose, or when using a multidrug regimen, it can be beneficial to eliminate one drug or to switch classes, for instance, from calcineurin inhibitors, which confer a higher risk, to mTOR inhibitors, which confer a lower risk (63). However, the decreased risk of cutaneous malignancy associated with sirolimus is balanced by an increased risk of serious adverse effects. The most common adverse events are edema, acneiform eruption, aphthous ulcers, and proteinuria (64). The risk–benefit ratio improves with lower doses of sirolimus and a low conversion rate from calcineurin inhibitors (65). Finally, immunotherapy with immune checkpoint inhibitors (ICIs) could represent a potential systemic therapeutic approach for the treatment of NMSCs in advanced and metastatic stages. However, since immunotherapy is not effective in all patients and can possibly induce severe adverse effects, a central clinical question is how to correctly recognize those patients who could be proper candidates for this therapeutic option. To this purpose, Zelin et al., (66) have recently reviewed the potential features and biomarkers used to predict the outcome of ICIs therapy for NMSCs in both general population and in organ transplant recipients. It is likely that time from transplantation and choice of immunosuppression could play a major role in defining the outcome in patients undergoing immunotherapy, although Abdel-Wahab et al. found no significant correlation between these factors (67). Additional studies are needed to address this important topic.

## 5. Prevention

Increased emphasis on a proactive rather than reactive approach to skin cancer is starting to show benefits for patient care (68). An Australian survey showed that renal transplant patients were more compliant with sun protection strategies than the general population (69), and regular and correct application of high sun protection factor sunscreen has demonstrated a reduced incidence of NMSC in transplanted patients (70). Early treatment of precancerous lesions (especially AKs) with cryotherapy or topical application of imiquimod or diclofenac is recommended (58). Photodynamic therapy with aminolevulinic acid is effective, well tolerated and is associated with a good cosmetic outcome (71). Cutaneous lesions that recur after non-surgical therapies need prompt biopsy or, if possible, radical excision, to rule out an NMSC. This strongly supports recommendations from SCOPE (Skin Care in Organ Transplant Patients, Europe) and ITSCC (International Transplant Skin Cancer Collaborative) that all solid organ transplant recipients, should be regularly followed up in a specialist dermatologist clinic to enhance early detection of lesions (72).

## 6. Chemoprophylaxis

Oral acitretin significantly decreased the rates of AKs and SCCs in the renal transplant and general population. Adverse effects are dose-related and include mucocutaneous xerosis, liver toxicity, arthralgia/myalgia (58, 73). Retinoids should be administered for many years, and rebound NMSC development on cessation may occur (58, 73). Nicotinamide 500 mg twice daily provides protection against photocarcinogenesis in the general population at high risk of NMSC, reducing the incidence of AKs and NMSCs by 13 and 23% (74), but with no general agreement (74). As nicotinamide is well tolerated and has few side effects, we propose that nicotinamide should be administered to all patients at risk of NMSC and actinic keratoses.

## Author contributions

GT, SG, and GZ conceived and wrote this article. GS contributed to literature search and revised the manuscript. All authors contributed to the article and approved the submitted version.

## Conflict of interest

The authors declare that the research was conducted in the absence of any commercial or financial relationships that could be construed as a potential conflict of interest.

## Publisher’s note

All claims expressed in this article are solely those of the authors and do not necessarily represent those of their affiliated organizations, or those of the publisher, the editors and the reviewers. Any product that may be evaluated in this article, or claim that may be made by its manufacturer, is not guaranteed or endorsed by the publisher.

## References

[1] Cooper JE. Evaluation and treatment of acute rejection in kidney allografts. Clin J Am Soc Nephrol. (2020) 15:430–8. doi: 10.2215/CJN.11991019, PMID: 32066593 PMC7057293

[2] Caletti C Manuel Ferraro P Corvo A Tessari G Sandrini S Capelli I. Impact of 3 major maintenance immunosuppressive protocols on long-term clinical outcomes: result of a large multicenter Italian cohort study including 5635 renal transplant recipients. Transplant Proc. (2019) 51:136–9. doi: 10.1016/j.transproceed.2018.02.209, PMID: 30655148

[3] Mehta R Cherukuri A Sood P Puttarajappa C Hoffman W Wu C. Kidney transplant survival: transforming early post-transplant opportunities to long-term success. Clin Transpl. (2015) 31:227–37. PMID: 28514584

[4] Wan SS Ying TD Wyburn K Roberts DM Wyld M Chadban SJ. The treatment of antibody-mediated rejection in kidney transplantation: an updated systematic review and meta-analysis. Transplantation. (2018) 102:557–68. doi: 10.1097/TP.0000000000002049 29315141

[5] Wu C Evans I Joseph R Shapiro R Tan H Basu A. Comorbid conditions in kidney transplantation: association with graft and patient survival. J Am Soc Nephrol. (2005) 16:3437–44. doi: 10.1681/ASN.2005040439 16176999

[6] Wu DA Robb ML Forsythe JLR Bradley C Cairns J Draper H. Recipient comorbidity and survival outcomes after kidney transplantation: a UK-wide prospective cohort study. Transplantation. (2020) 104:1246–55. doi: 10.1097/TP.0000000000002931, PMID: 31449188

[7] Ulrich C Kanitakis J Stockfleth E Euvrard S. Skin cancer in organ transplant recipients--where do we stand today? Am J Transplant. (2008) 8:2192–8. doi: 10.1111/j.1600-6143.2008.02386.x, PMID: 18782290

[8] Ciążyńska M Kamińska-Winciorek G Lange D Lewandowski B Reich A Sławińska M. The incidence and clinical analysis of non-melanoma skin cancer. Sci Rep. (2021) 11:4337. doi: 10.1038/s41598-021-83502-8, PMID: 33619293 PMC7900109

[9] Iannacone MR Sinnya S Pandeya N Isbel N Campbell S Fawcett J. Prevalence of skin cancer and related skin tumors in high-risk kidney and liver transplant recipients in Queensland. J Invest Dermatol. (2016) 136:1382–6. doi: 10.1016/j.jid.2016.02.804, PMID: 26968258

[10] Jiyad Z O'Rourke P Soyer HP Green AC. Actinic keratosis-related signs predictive of squamous cell carcinoma in renal transplant recipients: a nested case-control study. Br J Dermatol. (2017) 176:965–70. doi: 10.1111/bjd.15019, PMID: 27584866

[11] Euvrard S Kanitakis J Claudy A. Skin cancers after organ transplantation. N Engl J Med. (2003) 348:1681–91. doi: 10.1056/NEJMra022137 12711744

[12] Berg D Otley CC. Skin cancer in organ transplant recipients: epidemiology, pathogenesis, and management. J Am Acad Dermatol. (2002) 47:1–20. doi: 10.1067/mjd.2002.125579 12077575

[13] Bouwes Bavinck JN Hardie DR Green A Cutmore S MacNaught A O'Sullivan B. The risk of skin cancer in renal transplant recipients in Queensland, Australia. A follow-up study. Transplantation. (1996) 61:715–21. doi: 10.1097/00007890-199603150-00008 8607173

[14] Tessari G Naldi L Boschiero L Minetti E Sandrini S Nacchia F. Incidence of primary and second cancers in renal transplant recipients: a multicenter cohort study. Am J Transplant. (2013) 13:214–21. doi: 10.1111/j.1600-6143.2012.04294.x, PMID: 23057816

[15] Tessari G Naldi L Boschiero L Nacchia F Fior F Forni A. Incidence and clinical predictors of a subsequent nonmelanoma skin cancer in solid organ transplant recipients with a first nonmelanoma skin cancer: a multicenter cohort study. Arch Dermatol. (2010) 146:294–9. doi: 10.1001/archdermatol.2009.377, PMID: 20231501

[16] Lindelöf B Sigurgeirsson B Gäbel H Stern RS. Incidence of skin cancer in 5356 patients following organ transplantation. Br J Dermatol. (2000) 143:513–9. PMID: 10971322

[17] Euvrard S Kanitakis J Pouteil-Noble C Dureau G Touraine JL Faure M. Comparative epidemiologic study of premalignant and malignant epithelial cutaneous lesions developing after kidney and heart transplantation. J Am Acad Dermatol. (1995) 33:222–9. doi: 10.1016/0190-9622(95)90239-2, PMID: 7622649

[18] Ferrándiz C Fuente MJ Ribera M Bielsa I Fernández MT Lauzurica R. Epidermal dysplasia and neoplasia in kidney transplant recipients. J Am Acad Dermatol. (1995) 33:590–6. doi: 10.1016/0190-9622(95)91276-2, PMID: 7673490

[19] Koljonen V Sahi H Böhling T Mäkisalo H. Post-transplant Merkel Cell Carcinoma. Acta Derm Venereol. (2016) 96:442–7. doi: 10.2340/00015555-2284, PMID: 26554531

[20] Clarke CA Robbins HA Tatalovich Z Lynch CF Pawlish KS Finch JL. Risk of merkel cell carcinoma after solid organ transplantation. J Natl Cancer Inst. (2015) 107:dju382. doi: 10.1093/jnci/dju382 25575645 PMC4311175

[21] Ascha M Ascha MS Tanenbaum J Bordeaux JS. Risk factors for melanoma in renal transplant recipients. JAMA Dermatol. (2017) 153:1130–6. doi: 10.1001/jamadermatol.2017.2291, PMID: 28746700 PMC5710441

[22] Piselli P Serraino D Segoloni GP Sandrini S Piredda GB Scolari MP. Immunosuppression and cancer study group. Risk of de novo cancers after transplantation: results from a cohort of 7217 kidney transplant recipients, Italy 1997-2009. Eur J Cancer. (2013) 49:336–44. doi: 10.1016/j.ejca.2012.09.013, PMID: 23062667

[23] Vena GA Fargnoli MC Cassano N Argenziano G. Drug-induced eruptive melanocytic nevi. Expert Opin Drug Metab Toxicol. (2017) 13:293–300. doi: 10.1080/17425255.2017.1247155, PMID: 27759434

[24] Koseoglu G Akay BN Kucuksahin O Erdem C. Dermoscopic changes in melanocytic nevi in patients receiving immunosuppressive and biologic treatments: results of a prospective case-control study. J Am Acad Dermatol. (2015) 73:623–9. doi: 10.1016/j.jaad.2015.07.013, PMID: 26293825

[25] Piaserico S Alaibac M Fortina AB Peserico A. Clinical and dermatoscopic fading of post-transplant eruptive melanocytic nevi after suspension of immunosuppressive therapy. J Am Acad Dermatol. (2006) 54:338–40. doi: 10.1016/j.jaad.2005.06.023, PMID: 16443071

[26] Hafner J Kunzi W Weinreich T. Malignant fibrous histiocytoma and atypical fibroxanthoma in renal transplant recipients. Dermatology. (1999) 198:29–32. doi: 10.1159/000018060, PMID: 10026398

[27] Wehrli BM Janzen DL Shokeir O Masri BA Byrne SK O'Connell JX. Epithelioid angiosarcoma arising in a surgically constructed arteriovenous fistula: a rare complication of chronic immunosuppression in the setting of renal transplantation. Am J Surg Pathol. (1998) 22:1154–9. doi: 10.1097/00000478-199809000-00016, PMID: 9737250

[28] Kibe Y Kishimoto S Katoh N Yasuno H Yasumura T Oka T. Angiosarcoma of the scalp associated with renal transplantation. Br J Dermatol. (1997) 136:752–6. doi: 10.1046/j.1365-2133.1997.6691611.x, PMID: 9205512

[29] Tufaro AP Azoury SC Crompton JG Straughan DM Reddy S Prasad NB. Rising incidence and aggressive nature of cutaneous malignancies after transplantation: an update on epidemiology, risk factors, management and surveillance. Surg Oncol. (2015) 24:345–52. doi: 10.1016/j.suronc.2015.09.007, PMID: 26690824

[30] Jensen P Hansen S Møller B Leivestad T Pfeffer P Geiran O. Skin cancer in kidney and heart transplant recipients and different long-term immunosuppressive therapy regimens. J Am Acad Dermatol. (1999) 40:177–86. doi: 10.1016/S0190-9622(99)70185-4, PMID: 10025742

[31] Ingvar A Smedby KE Lindelöf B Fernberg P Bellocco R Tufveson G. Immunosuppressive treatment after solid organ transplantation and risk of post-transplant cutaneous squamous cell carcinoma. Nephrol Dial Transplant. (2010) 25:2764–71. doi: 10.1093/ndt/gfp425, PMID: 19729465

[32] O'Donovan P Perrett CM Zhang X Montaner B Xu YZ Harwood CA. Azathioprine and UVA light generate mutagenic oxidative DNA damage. Science. (2005) 309:1871–4. doi: 10.1126/science.1114233, PMID: 16166520 PMC2426755

[33] Geissler EK. Post-transplantation malignancies: here today, gone tomorrow? Nat Rev Clin Oncol. (2015) 12:705–17. doi: 10.1038/nrclinonc.2015.186, PMID: 26483298

[34] Jensen P Hansen S Møller B Leivestad T Pfeffer P Fauchald P. Are renal transplant recipients on CsA-based immunosuppressive regimens more likely to develop skin cancer than those on azathioprine and prednisolone? Transplant Proc. (1999) 31:1120. doi: 10.1016/S0041-1345(98)01928-9, PMID: 10083500

[35] Yarosh DB Pena AV Nay SL Canning MT Brown DA. Calcineurin inhibitors decrease DNA repair and apoptosis in human keratinocytes following ultraviolet B irradiation. J Invest Dermatol. (2005) 125:1020–5. doi: 10.1111/j.0022-202X.2005.23858.x, PMID: 16297204

[36] Kuschal C Thoms KM Schubert S Schäfer A Boeckmann L Schön MP. Skin cancer in organ transplant recipients: effects of immunosuppressive medications on DNA repair. Exp Dermatol. (2012) 21:2–6. doi: 10.1111/j.1600-0625.2011.01413.x, PMID: 22151386

[37] Kasiske BL Snyder JJ Gilbertson DT Wang C. Cancer after kidney transplantation in the United States. Am J Transplant. (2004) 4:905–13. doi: 10.1111/j.1600-6143.2004.00450.x 15147424

[38] Crespo-Leiro MG Alonso-Pulpón L Vázquez de Prada JA Almenar L Arizón JM Brossa V. Malignancy after heart transplantation: incidence, prognosis and risk factors. Am J Transplant. (2008) 8:1031–9. doi: 10.1111/j.1600-6143.2008.02196.x 18416739

[39] Molina BD Leiro MG Pulpón LA Mirabet S Yañez JF Bonet LA. Incidence and risk factors for nonmelanoma skin cancer after heart transplantation. Transplant Proc. (2010) 42:3001–5. doi: 10.1016/j.transproceed.2010.08.003, PMID: 20970593

[40] Krásová M Sečníková Z Göpfertová D Hercogová J Viklický O Jůzlová K. Immunosuppressive therapy in the posttransplant period and skin cancer. Dermatol Ther. (2016) 29:433–6. doi: 10.1111/dth.12379, PMID: 27328964

[41] Watorek E Boratynska M Smolska D Patrzalek D Klinger M. Malignancy after renal transplantation in the new era of immunosuppression. Ann Transplant. (2011) 16:14–8. doi: 10.12659/AOT.881859, PMID: 21716180

[42] Wimmer CD Angele MK Schwarz B Pratschke S Rentsch M Khandoga A. Impact of cyclosporine versus tacrolimus on the incidence of de novo malignancy following liver transplantation: a single center experience with 609 patients. Transpl Int. (2013t) 26:999–1006. doi: 10.1111/tri.12165, PMID: 23952102

[43] Coghill AE Johnson LG Berg D Resler AJ Leca N Madeleine MM. Immunosuppressive medications and squamous cell skin carcinoma: nested case-control study within the skin cancer after organ transplant (SCOT) cohort. Am J Transplant. (2016) 16:565–73. doi: 10.1111/ajt.13596, PMID: 26824445 PMC5500236

[44] Aguiar D Martínez-Urbistondo D D'Avola D Iñarrairaegui M Pardo F Rotellar F. Conversion from Calcineurin inhibitor-based immunosuppression to mycophenolate Mofetil in monotherapy reduces risk of De novo malignancies after liver transplantation. Ann Transplant. (2017) 22:141–7. doi: 10.12659/AOT.901556, PMID: 28302995 PMC12577489

[45] Piponniau L Kittleson M Patel J Rafiei M Osborne A Dhiantravan V. Mycophenolate not azathioprine is associated with increased risk for skin cancer after heart transplant. J Heart Lung Transpl. (2013) 32:S199. doi: 10.1016/j.healun.2013.01.490

[46] Wang YJ Chi NH Chou NK Huang SC Wang CH Wu IH. Malignancy after heart transplantation under Everolimus versus mycophenolate Mofetil immunosuppression. Transplant Proc. (2016) 48:969–73. doi: 10.1016/j.transproceed.2015.12.071, PMID: 27234781

[47] Knoll GA Kokolo MB Mallick R Beck A Buenaventura CD Ducharme R. Effect of sirolimus on malignancy and survival after kidney transplantation: systematic review and meta-analysis of individual patient data. BMJ. (2014) 349:g6679. doi: 10.1136/bmj.g6679, PMID: 25422259 PMC4241732

[48] Ying T Wong G Lim W Kanellis J Pilmore H Campbell S. De novo or early conversion to everolimus and long-term cancer outcomes in kidney transplant recipients: a trial-based linkage study. Am J Transplant. (2018) 18:2977–86. doi: 10.1111/ajt.14948 29802791

[49] Dantal J Morelon E Rostaing L Goffin E Brocard A Tromme I. Sirolimus for secondary prevention of skin cancer in kidney transplant recipients: 5-year results. J Clin Oncol. (2018) 36:2612–20. doi: 10.1200/JCO.2017.76.6691, PMID: 30016177

[50] Hufbauer M Akgül B. Molecular mechanisms of human papillomavirus induced skin carcinogenesis. Viruses. (2017) 9:187. doi: 10.3390/v9070187, PMID: 28708084 PMC5537679

[51] Chin-Hong PV Reid GE. AST infectious diseases Community of Practice. Human papillomavirus infection in solid organ transplant recipients: guidelines from the American Society of Transplantation infectious diseases Community of Practice. Clin Transpl. (2019) 33:e13590. doi: 10.1111/ctr.13590, PMID: 31077438

[52] Weissenborn SJ Nindl I Purdie K Harwood C Proby C Breuer J. Human papillomavirus-DNA loads in actinic keratoses exceed those in non-melanoma skin cancers. J Invest Dermatol. (2005) 125:93–7. doi: 10.1111/j.0022-202X.2005.23733.x, PMID: 15982308

[53] Harwood CA Surentheran T McGregor JM Spink PJ Leigh IM Breuer J. Human papillomavirus infection and non-melanoma skin cancer in immunosuppressed and immunocompetent individuals. J Med Virol. (2000) 61:289–97. doi: 10.1002/1096-9071(200007)61:3<289::AID-JMV2>3.0.CO;2-Z, PMID: 10861635

[54] Stockfleth E Nindl I Sterry W Ulrich C Schmook T Meyer T. Human papillomaviruses in transplant-associated skin cancers. Dermatol Surg. (2004) 30:604–9. doi: 10.1111/j.1524-4725.2004.00144.x, PMID: 15061843

[55] Reuschenbach M Tran T Faulstich F Hartschuh W Vinokurova S Kloor M. High-risk human papillomavirus in non-melanoma skin lesions from renal allograft recipients and immunocompetent patients. Br J Cancer. (2011) 104:1334–41. doi: 10.1038/bjc.2011.95, PMID: 21427726 PMC3078602

[56] Proby CM Harwood CA Neale RE Green AC Euvrard S Naldi L. EPI-HPV-UV-CA group. A case-control study of betapapillomavirus infection and cutaneous squamous cell carcinoma in organ transplant recipients. Am J Transplant. (2011) 11:1498–508. doi: 10.1111/j.1600-6143.2011.03589.x, PMID: 21718442

[57] O'Reilly Zwald F Brown M. Skin cancer in solid organ transplant recipients: advances in therapy and management: part I. epidemiology of skin cancer in solid organ transplant recipients. J Am Acad Dermatol. (2011) 65:253–61. doi: 10.1016/j.jaad.2010.11.062, PMID: 21763561

[58] Howard MD Su JC Chong AH. Skin cancer following solid organ transplantation: a review of risk factors and models of care. Am J Clin Dermatol. (2018) 19:585–97. doi: 10.1007/s40257-018-0355-8, PMID: 29691768

[59] Xiol X Guardiola J Menendez S Lama C Figueras J Marcoval J. Risk factors for development of de novo neoplasia after liver transplantation. Liver Transpl. (2001) 7:971–5. doi: 10.1053/jlts.2001.28744 11699033

[60] Peris K Alaibac M Argenziano G Di Stefani A Fargnoli MC Frascione P. Cutaneous squamous cell carcinoma. Italian guidelines by SIDeMaST adapted to and updating EADO/EDF/EORTC guidelines. G Ital Dermatol Venereol. (2018) 153:747–62. doi: 10.23736/S0392-0488.18.06093-5, PMID: 29898593

[61] Veness MJ Delishaj D Barnes EA Bezugly A Rembielak A. Current role of radiotherapy in non-melanoma skin cancer. Clin Oncol (R Coll Radiol). (2019) 31:749–58. doi: 10.1016/j.clon.2019.08.004 31447088

[62] Otley CC Berg D Ulrich C Stasko T Murphy GM, Salasche SJ Salasche SJ Christenson LJ Sengelmann R Loss GE JR Garces J Reduction Of Immunosuppression Task Force Of The International Transplant Skin Cancer Collaborative And The Skin Care In Organ Transplant Patients Europe; Reduction Of Immunosuppression Task Force Of The International Transplant Skin Cancer Collaborative And The Skin Care In Organ Transplant Patients Europe. Reduction of immunosuppression for transplant-associated skin cancer: expert consensus survey. Br J Dermatol (2006);154:395–400, doi: 10.1111/j.1365-2133.2005.07087.x, PMID: . 16445766

[63] Colegio OR Hanlon A Olasz EB Carucci JA. Sirolimus reduces cutaneous squamous cell carcinomas in transplantation recipients. J Clin Oncol. (2013) 31:3297–8. doi: 10.1200/JCO.2013.50.6840, PMID: 23918945

[64] Zaza G Granata S Tomei P Masola V Gambaro G Lupo A. mTOR inhibitors and renal allograft: yin and Yang. J Nephrol. (2014) 27:495–506. doi: 10.1007/s40620-014-0103-y, PMID: 24804854

[65] Zaza G Tomei P Ria P Granata S Boschiero L Lupo A. Systemic and nonrenal adverse effects occurring in renal transplant patients treated with mTOR inhibitors. Clin Dev Immunol. (2013) 2013:403280:1–13. doi: 10.1155/2013/403280 24151517 PMC3789319

[66] Zelin E Maronese CA Dri A Toffoli L Di Meo N Nazzaro G. Identifying candidates for immunotherapy among patients with non-melanoma skin cancer: a review of the potential predictors of response. J Clin Med. (2022) 11:3364. doi: 10.3390/jcm11123364, PMID: 35743435 PMC9225110

[67] Abdel-Wahab N Safa H Abudayyeh A Johnson DH Trinh VA Zobniw CM. Checkpoint inhibitor therapy for cancer in solid organ transplantation recipients: an institutional experience and a systematic review of the literature. J Immunother Cancer. (2019) 7:106. doi: 10.1186/s40425-019-0585-1, PMID: 30992053 PMC6469201

[68] Otley CC. Organization of a specialty clinic to optimize the care of organ transplant recipients at risk for skin cancer. Dermatol Surg. (2000) 26:709–12. doi: 10.1046/j.1524-4725.2000.00091.x, PMID: 10886290

[69] Leung VKY Dobbinson SJ Goodman DJ Kanellis J Chong AH. Skin cancer history, sun-related attitudes, behaviour and sunburn among renal transplant recipients versus general population. Australas J Dermatol. (2018) 59:e106–13. doi: 10.1111/ajd.12591, PMID: 28332195

[70] Ulrich C Jürgensen JS Degen A Hackethal M Ulrich M Patel MJ. Prevention of non-melanoma skin cancer in organ transplant patients by regular use of a sunscreen: a 24 months, prospective, case-control study. Br J Dermatol. (2009) 161:78–84. doi: 10.1111/j.1365-2133.2009.09453.x, PMID: 19775361

[71] O'Reilly Zwald F Brown M. Skin cancer in solid organ transplant recipients: advances in therapy and management: part II. Management of skin cancer in solid organ transplant recipients. J Am Acad Dermatol. (2011) 65:263–79. doi: 10.1016/j.jaad.2010.11.063, PMID: 21763562

[72] Martinez JC Otley CC Stasko T Euvrard S Brown C Schanbacher CF. Transplant-skin cancer collaborative. Defining the clinical course of metastatic skin cancer in organ transplant recipients: a multicenter collaborative study. Arch Dermatol. (2003) 139:301–6. doi: 10.1001/archderm.139.3.301, PMID: 12622621

[73] Herold M Good AJ Nielson CB Longo MI. Use of topical and systemic Retinoids in solid organ transplant recipients: update and review of the current literature. Dermatol Surg. (2019) 45:1442–9. doi: 10.1097/DSS.0000000000002072, PMID: 31403546

[74] Damian DL. Nicotinamide for skin cancer chemoprevention. Australas J Dermatol. (2017) 58:174–80. doi: 10.1111/ajd.12631 28321860

